# PARK2-Mediated PGK1 Degradation Suppresses Partial Epithelial-Mesenchymal Transition and Metastasis in Triple-Negative Breast Cancer

**DOI:** 10.32604/or.2026.081209

**Published:** 2026-06-16

**Authors:** Zhengzheng Li, Haitong Xie, Yujuan Chen, Qiuyan Li, Xing Yuan, Xinyue Dai, Jie Chen

**Affiliations:** 1 Department of General Surgery, West China Hospital, Sichuan University, Chengdu, China; 2 Breast Center, West China Hospital, Sichuan University, Chengdu, China; 3 Breast Center, West China Tianfu Hospital, Sichuan University, Chengdu, China; 4 Department of Pulmonary and Critical Care Medicine, West China Hospital, Sichuan University, Chengdu, China

**Keywords:** Triple-negative breast cancer (TNBC), phosphoglycerate kinase 1 (PGK1), Parkinson disease protein 2 (PARK2), proteasomal degradation, epithelial-to-mesenchymal transition, metastasis

## Abstract

**Objectives**: Triple-negative breast cancer (TNBC) is an aggressive subtype lacking targeted therapies. Phosphoglycerate kinase 1 (PGK1) drives TNBC progression, but mechanisms governing its protein stability remain unclear. This study aims to identify the E3 ubiquitin ligase responsible for PGK1 degradation and evaluate its therapeutic potential against metastasis. **Methods:** Clinical datasets and 50 human TNBC tissues were analyzed via multiplex immunohistochemistry. Co-immunoprecipitation, ubiquitination linkage assays, and structural modeling were utilized for *in vitro* mechanistic studies in TNBC cells. Additionally, functional impacts on epithelial-mesenchymal transition (EMT) and metastasis were evaluated using transwell assays and an *in vivo* mouse lung metastasis model. **Results:** Parkinson disease protein 2 (PARK2) is a novel E3 ubiquitin ligase that mediates proteasomal degradation of PGK1 in TNBC cells. Elevated *PGK1* expression and reduced *PARK2* expression in TNBC, with high *PGK1* levels correlating with unfavorable overall survival (HR: 2.138, 95%CI:1.001 to 4.569, *p* = 0.049). PARK2 physically binds PGK1 via its RING2 domain and promotes K48-linked polyubiquitination, leading to proteasomal degradation. A significant negative correlation between PARK2 and PGK1 at the protein levels were confirmed in 50 TNBC tumor tissues (Spearman’s rho = −0.58, *p* < 0.001). Functionally, *PARK2* overexpression reduced mesenchymal markers (Vimentin, Snail1, Slug) and suppressed migration and invasion of TNBC cells, effects that were reversed by *PGK1* overexpression. PARK2 significantly inhibited PGK1-mediated lung metastasis in *in vivo* tail vein injection models **Conclusion:** These findings establish the PARK2-PGK1 axis as a critical regulator of partial epithelial-mesenchymal transition and metastasis in TNBC, suggesting that strategies to enhance PARK2 expression or activity may represent promising therapeutic approaches for this aggressive breast cancer subtype.

## 1 Introduction

Globally, breast cancer is the tumor with the highest incidence and mortality rates among women [1]. It exhibits a wide range of molecular subtypes and is characterized by high heterogeneity. Based on the protein-level expression of estrogen receptor (ER), progesterone receptor (PR), and receptor tyrosine kinase HER2/ERBB2, breast tumors can be roughly classified into four subtypes: Luminal A, Luminal B, HER2-overexpressing, and triple-negative breast cancer (TNBC) [2]. Among these, approximately 80% of TNBCs are basal-like subtype [3]. TNBC represents the most aggressive breast cancer subtype and remains refractory to receptor-targeted therapies [4,5,6]. For patients who are not eligible for surgery, radiotherapy and chemotherapy remain the main treatment options [7,8]. The prognosis remains poor due to frequent metastasis and therapeutic resistance, accounting for a disproportionate number of breast cancer deaths [9]. To overcome these challenges, current research is pivoting toward identifying novel molecular vulnerabilities and developing advanced therapeutic platforms [10,11,12]. For instance, recent advancements in nanomedicine, such as the use of MXene nanoparticles, offer revolutionary potential for the precise delivery of therapeutic agents to refractory tumors [13,14]. However, the successful application of such emerging technologies relies heavily on the identification of specific, actionable molecular targets driving TNBC progression.

Phosphoglycerate kinase (PGK) functions as a key glycolytic enzyme that facilitates the transformation of 1,3-bisphosphoglycerate into 3-phosphoglycerate while producing ATP. The X-linked isoform PGK1 exhibits ubiquitous expression across diverse cell types and serves as a critical regulatory point for cellular energy generation and oxidation-reduction homeostasis in the latter stages of glycolysis [15]. While elevated PGK1 expression is a hallmark of breast cancer and correlates with poor prognosis [15,16,17], recent “state-of-the-art” studies suggest that its contribution to tumorigenesis extends far beyond simple metabolic flux. A growing body of evidence indicates that PGK1 possesses “moonlighting” functions—non-metabolic activities that drive malignancy through protein-protein interactions. For example, PGK1 has been shown to interact with HIF-2α to promote epithelial-mesenchymal transition (EMT) [18]. Crucially, studies have shown that while *PGK1* knockdown significantly inhibits metastasis [16,19,20], inhibiting its enzymatic activity alone often fails to produce the same effect, as metabolic plasticity allows tumor cells to shunt intermediates into alternative pathways like the pentose phosphate pathway [21].

This dichotomy presents a critical knowledge gap and a specific therapeutic argument: if the metastatic capability of PGK1 relies on its physical presence and protein interactions rather than solely its enzymatic output, then the most effective therapeutic strategy is not enzymatic inhibition, but rather the destabilization and degradation of the PGK1 protein itself. Targeted protein degradation via the ubiquitin-proteasome system (UPS) represents a promising frontier in cancer therapy [22], while it is known that PGK1 can undergo polyubiquitination and proteasomal degradation [17,23], the specific E3 ubiquitin ligase responsible for regulating PGK1 stability in the context of TNBC remains unidentified. Defining this regulatory mechanism is essential, as it would provide the molecular rationale for therapies that restore the endogenous degradation of this metastatic driver.

This study aims to address this gap by identifying the specific E3 ligase that mediates PGK1 degradation in TNBC. It was hypothesized that restoring this ubiquitin-mediated pathway will destabilize PGK1, thereby suppressing the non-metabolic signaling networks that drive invasion and metastasis.

## 2 Materials and Methods

### 2.1 Ethics Approval

This study was conducted in accordance with the ethical principles outlined in the Declaration of Helsinki and was approved by the Biomedical Ethics Review Committee of West China Hospital, Sichuan University (approval No. 20251821). All human tissue samples (primary TNBC tumor tissues) were obtained with informed consent from patients or their legal representatives. The use of these samples for immunohistochemistry analysis was explicitly covered under the approved protocol. Patient data were anonymized to ensure confidentiality.

For animal experiments, all procedures involving mice were performed in compliance with institutional guidelines for animal welfare and were approved by the same ethics committee as part of the overarching study protocol (approval No. 20251821).

### 2.2 Data Retrieval and Processing

Gene expression data were retrieved from the UCSC Xena Browser (https://xenabrowser.net/) [24]. The combined dataset included expression profiles from the Genotype-Tissue Expression (GTEx) project and the Cancer Genome Atlas Breast Cancer (TCGA-BRCA) cohort. For the TNBC subset, data from basal-like breast cancer samples were specifically extracted and analyzed. Single-cell RNA sequencing data were obtained from Wu et al.’s publication [25]. These data were accessible through The Single Cell Portal (https://singlecell.broadinstitute.org/single_cell).

Patients classified as the Basal-like subtype within the TCGA-BRCA cohort were identified based on the PAM50 gene signature. These patients were stratified into high and low *PGK1* expression groups using an optimal cutoff value, which was determined by calculating the maximum Youden’s index. Kaplan-Meier survival plots were generated and evaluated using the log-rank test. Progression-free interval (PFI) was defined as the time from diagnosis to the first event (recurrence, progression, or death), and overall survival was defined as the time from diagnosis to death from any cause.

Potential E3 ubiquitin ligases for PGK1 were predicted using the BioGRID database (https://thebiogrid.org/). Gene Set Enrichment Analysis (GSEA) was performed using the GSEA software (v4.3.2, Broad Institute, Cambridge, MA, USA) with the Hallmark gene sets from Molecular Signatures Database (MSigDB). Normalized enrichment scores (NES) and false discovery rates (FDR) were calculated to assess the significance of enrichment. Gene sets with a FDR < 0.25 and a nominal *p*-value < 0.05 were considered significantly enriched.

### 2.3 Cell Culture

MDA-MB-231 cells were purchased from Procell (Wuhan, Hubei, China) and were cultured in Dulbecco’s Modified Eagle Medium (DMEM) (Procell, PM150210) supplemented with 10% fetal bovine serum (FBS, Procell, 164210) and 1% penicillin-streptomycin (P/S) solution (Procell, PB180120) at 37°C in a humidified atmosphere of 5% CO_2_.

BT-549 cells were purchased from Procell (Wuhan) and were cultured in Roswell Park Memorial Institute (RPMI)-1640 medium (Procell, PM150110) supplemented with 10% FBS (Procell, 164210), 10 μg/mL Insulin (Procell, PB180432), and 1% P/S solution (Procell, PB180120) at 37°C in a humidified atmosphere of 5% CO_2_.

Both cell lines were authenticated by the supplier using short tandem repeat (STR) profiling prior to use. Furthermore, all cell cultures were routinely tested for mycoplasma contamination and confirmed to be negative.

Although these cell lines are classified as the Claudin-low subtype, they were selected for this study as they exhibit pronounced mesenchymal phenotypes and are widely established models for investigating EMT-driven metastasis in aggressive TNBC, thereby serving as suitable *in vitro* counterparts to the poor-prognosis Basal-like clinical cohort.

### 2.4 Lentivirus Preparation and Overexpression

The human PARK2 gene (NM_004562) and human PGK1 gene (NM_000291) were cloned into the pLVX-puro vector (Takara Bio, 632164, Beijing, China) using EcoRI and BamHI restriction sites to generate the *PARK2* overexpression construct (PARK2-OE) and *PGK1* overexpression construct (PGK1-OE), respectively. All generated constructs were verified by Sanger sequencing to confirm sequence integrity prior to use. For transfection, cells were seeded in 6-well plates at a density of 3 × 10^5^ cells per well. Upon reaching 70–80% confluency, cells were transfected with 2.5 μg of PARK2-OE or an empty vector as control using Lipofectamine 3000 (Thermo Fisher Scientific, L3000015, Foster City, CA, USA) at a 1:2 ratio (μg DNA to μL Lipofectamine 3000) according to the manufacturer’s instructions. For ubiquitination assays, cells were co-transfected with PARK2 constructs and HA-tagged ubiquitin (#18712, Addgene, Watertown, MA, USA), pCMV-HA-Ub-K48R, or pCMV-HA-Ub-K63R (maintaining a total of 2.5 μg DNA per well).

To examine the structural requirements for PARK2 binding to PGK1, Flag-tagged deletion mutants of PARK2 were constructed. The full-length PARK2 (amino acids 1-465) and a deletion mutant PARK2 (amino acids 1-414) with C-terminal Flag tag were generated using pLVX-3 × Flag-Puro vector (Solarbio, VT012670, Beijing, China). Lentivirus packaging was conducted by co-transfecting the recombinant transfer vector, the packaging plasmid (psPAX2, #12260, Addgene), and the envelope plasmid (pMD2.G, #12259, Addgene) into HEK293T cells. Supernatants containing lentiviral particles were collected 48 and 72hours post-transfection. MDA-MB-231 and BT549 cells were infected with Flag-tagged PARK2 lentivirus for overexpression, at a multiplicity of infection (MOI) of 10. Stable clones were selected using 2 μg/mL puromycin (MCE, HY-B1743A, purity: 99.93%, Monmouth Junction, NJ, USA) for 2 weeks. Overexpression was validated by qRT-PCR and western blotting.

### 2.5 Structural Modeling

To illustrate the structural interface between PARK2 and PGK1, AlphaFold 3, a state-of-the-art protein structure prediction tool was employed. The structural models of PARK2 (Uniprot ID: O60260) and PGK1 (Uniprot ID: P00558) were generated and visualized using PyMOL software (v.3.1.0, Schrödinger, LLC, New York, NY, USA). The highlighted residues on PARK2 involved in the interaction (T414, T415, P424, and R442) were identified in the models. The RING0, RING1, and RING2 domains of PARK2 were annotated based on a previous PARK2 structural publication [26].

### 2.6 siRNA Transfection

siRNAs targeting human NEDD4 (NM_198400) and non-targeting control siRNAs were synthesized (sequences detailed below). Cells at 60–70% confluency were transfected with siRNAs at a final concentration of 50 nM using Lipofectamine RNAiMAX (Thermo Fisher Scientific, 13778030) according to the manufacturer’s protocol. Cells were incubated for 48 to 72 h post-transfection prior to harvesting for downstream assays. The following siRNA sequences were used:

NEDD4 siRNA-2800: 5′-GGAAGAAUCUUCUACAUAATT-3′ (sense), 5′-UUAUGUAGAAGAUUCUUCCTT-3′ (antisense);

NEDD4 siRNA-3116: 5′-GGUUCUUCCUGAUCUCAAATT-3′ (sense), 5′-UUUGAGAUCAGGAAGAACCTT-3′ (antisense);

NEDD4 siRNA-1129: 5′-CGACCGACUUCUCCAACAATT-3′ (sense), 5′-UUGUUGGAGAAGUCGGUCGTT-3′ (antisense);

Negative control siRNA: 5′-UUCUCCGAACGUGUCACGUTT-3′ (sense), 5′-ACGUGACACGUUCGGAGAATT-3′ (antisense).

Carboxyfluorescein (FAM)-negative control siRNA: 5′-UUCUCCGAACGUGUCACGUTT-3′ (sense), 5′-ACGUGACACGUUCGGAGAATT-3′ (antisense), which utilizes the identical sequence as the negative control but is modified with a FAM fluorescent tag to allow for visual monitoring of transfection efficiency. Knockdown efficiency was validated by qRT-PCR and Western blotting.

### 2.7 Quantitative Real-Time Polymerase Chain Reaction (qRT-PCR)

Total RNA was extracted using TRIzol reagent (Thermo Fisher Scientific, 15596026). RNA concentration and purity were assessed using a NanoDrop spectrophotometer by measuring the A260/A280 ratio (acceptable range 1.8–2.0), and RNA integrity was confirmed by 1% agarose gel electrophoresis. The extracted RNA was then reverse transcribed to cDNA using the SuperScript III First-Strand Synthesis System (Thermo Fisher Scientific, 18080051). qRT-PCR was performed using SYBR Green Master Mix (Thermo Fisher Scientific, 4309155) on an ABI 7500 Fast Real-Time PCR System (Thermo Fisher Scientific, 4351107). The qPCR reactions were prepared in a total volume of 20 μL, consisting of 10 μL of 2× SYBR Green Master Mix, 0.4 μM of each forward and reverse primer, and 2 μL of diluted cDNA template (equivalent to 20 ng of input RNA). No-template controls (NTCs) were included in every assay plate to monitor for potential reagent contamination. The thermal cycling conditions included an initial denaturation step at 95°C for 10 min, followed by 40 cycles of denaturation at 95°C for 15 s and a combined annealing/extension step at 60°C for 1 min. A melt curve analysis was performed at the end of each run to verify primer specificity. The relative mRNA expression levels were calculated using the 2^−^^ΔΔCt^ method, with GAPDH as an internal control. All qRT-PCR analyses were performed using three independent biological replicates, with each sample run in technical triplicates. The primers used are listed below:

*PARK2*:
Forward: 5′-CCAGAGGAAAGTCACCTGCGAA-3′;
Reverse: 5′-CTGAGGCTTCAAATACGGCACTG-3′;
*NEDD4*:
Forward: 5′-CAGAAGAGGCAGCTTACAAGCC-3′;
Reverse: 5′-CTTCCCAACCTGGTGGTAATCC-3′;
*PGK1*:
Forward: 5′-CCGCTTTCATGTGGAGGAAGAAG-3′;
Reverse: 5′-CTCTGTGAGCAGTGCCAAAAGC-3′;
*GAPDH*:
Forward: 5′-GTCTCCTCTGACTTCAACAGCG-3′;
Reverse: 5′-ACCACCCTGTTGCTGTAGCCAA-3′.

### 2.8 Western Blot Analysis

Cells were harvested and lysed in ice-cold RIPA buffer supplemented with protease and phosphatase inhibitors (Roche, 04693159001, Basel, Switzerland). Protein concentrations were determined using the BCA Protein Assay Kit (Thermo Fisher Scientific, 23227). Equal amounts (30 μg) of protein per lane were separated by 10% SDS-PAGE and transferred onto PVDF membranes (Millipore, IPVH00010, Billerica, MA, USA). Membranes were blocked with 5% non-fat milk in TBS-T for 1 h at room temperature and incubated overnight at 4°C with primary antibodies against PARK2 (1:2000, Proteintech, 66674-1-Ig, Wuhan, China), NEDD4 (1:2000, Proteintech, 21698-1-AP), PGK1 (1:2000, Proteintech [Rabbit], 17811-1-AP; 1:2000, Proteintech [Mouse], 68035-1-Ig), E-cadherin (1:1000, Proteintech, 20874-1-AP), Vimentin (1:2000, Proteintech, 10366-1-AP), N-cadherin (1:1000, Proteintech, 22018-1-AP), Snail1 (1:1000, Proteintech, 13099-1-AP), Slug (1:1000, Proteintech, 12129-1-AP), Twist1 (1:1000, Proteintech, 25465-1-AP) and ZEB1 (1:1000, Proteintech, 21544-1-AP) and β-actin (1:5000, Proteintech, 66009-1-Ig). Following three washes in TBS-T, membranes were incubated with HRP-conjugated secondary antibodies (Goat anti-Rabbit IgG, 1:5000, Proteintech, SA00001-2; Goat anti-Mouse IgG, 1:5000, Proteintech, SA00001-1) for 1 h at room temperature. After final washing, protein bands were visualized using an enhanced chemiluminescence (ECL) substrate (P0018AS, Beyotime, Shanghai, China), under automated exposure conditions on a Tanon 5200multi imaging system (Tanon, Shanghai, China). Band intensity was quantified using ImageJ software (version 1.54, National Institutes of Health, Bethesda, MD, USA). Target protein band intensities were normalized to their corresponding β-actin internal control bands to account for variations in protein loading.

### 2.9 Cycloheximide (CHX) Chase Assay

Cycloheximide (CHX) chase assays were performed to assess the protein stability of PGK1. Cells were treated with 50 μg/mL CHX (MCE, HY-12320, purity: 99.82%) with or without the proteasome inhibitor MG132 (5 μM, MCE, HY-13259, purity: 99.90%) for the indicated times (0, 2, 4, 6, and 8 h). Cells were harvested, and protein levels of PGK1 were analyzed by western blotting as described above. All CHX chase experiments were performed in three independent biological replicates. Protein band intensities were quantified using ImageJ software (version 1.54, National Institutes of Health) and normalized to β-actin (1:5000, Proteintech, 66009-1-Ig).

### 2.10 Immunofluorescence Staining

Immunofluorescence staining was performed to examine co-localization of PARK2 and PGK1 in MDA-MB-231 and BT549 cells. Cells were seeded on coverslips at a density of 1 × 10^5^ cells per well in 24-well plates and cultured until reaching 60–70% confluency prior to fixation. Cells were fixed in 4% paraformaldehyde for 15 min, permeabilized with 0.1% Triton X-100, and blocked with 5% BSA in PBS for 1 h at room temperature. Cells were incubated overnight at 4°C with primary antibodies against PARK2 (1:200, Proteintech, 66674-1-Ig) and PGK1 (1:200, Proteintech, 17811-1-AP), or Vimentin (1:200, Proteintech, 10366-1-AP). After washing, fluorescent secondary antibodies (Alexa Fluor 488-conjugated goat anti-mouse IgG, 1:500, Thermo Fisher Scientific, A28175; Alexa Fluor 555-conjugated goat anti-rabbit IgG, 1:500, Thermo Fisher Scientific, A-21428) were added and incubated at room temperature for 1 h. Nuclei were stained with DAPI (Thermo Fisher Scientific, D1306), and coverslips were mounted using ProLong Gold Antifade Mountant (Thermo Fisher Scientific, P36930). Images were captured using a fluorescence microscope (Zeiss Axio Imager A2, Carl Zeiss AG, Oberkochen, Germany).

### 2.11 Co-Immunoprecipitation (co-IP) Assays

To inhibit proteasomal degradation, cells were treated with 10 μM MG132 (Sigma-Aldrich, M7449, St. Louis, MO, USA) for 6 h before lysis for Co-IP assays. MDA-MB-231 and BT549 cells were lysed in ice-cold RIPA buffer (50 mM Tris-HCl, pH 7.4, 150 mM NaCl, 1% NP-40, 0.5% Sodium deoxycholate, 0.1% SDS, 1 mM EDTA) supplemented with protease and phosphatase inhibitors (Roche). Lysates were clarified by centrifugation at 12,000× *g* for 15 min at 4°C.

For Co-IP, a total volume of 500 μL containing 1000 μg of total protein was incubated with 4 μg of anti-PARK2 antibody (host: rabbit, Proteintech, 14060-1-AP) or anti-PGK1 antibody (host: rabbit, Proteintech, 17811-1-AP) overnight at 4°C. Parallel immunoprecipitations with an equivalent amount of normal rabbit IgG (Proteintech, 30000-0-AP) were performed under identical conditions to control for non-specific binding. Immune complexes were captured with Protein A/G Magnetic beads (Vazyme, PB101-01, Nanjing, Jiangsu, China) for 2 h at 4°C, washed extensively, and eluted in Laemmli buffer. Prior to immunoprecipitation, an aliquot of each whole cell lysate (input, representing 5% of the total protein used for IP) was saved for subsequent western blot analysis. Samples were then subjected to western blotting using anti-PARK2 (host: mouse, 1:2000, Proteintech, 66674-1-Ig), anti-PGK1 (host: mouse, 1:2000, Proteintech, 68035-1-Ig), anti-HA tag (host: mouse, 1:2000, Proteintech, 66006-2-Ig) or anti-ubiquitin (host: mouse, 1:1000, Cell Signaling Technology (CST), #3936, Danvers, MA, USA). For Co-IP with Flag-tagged proteins, cells were lysed and processed as described above, with the addition of 2 μg of anti-Flag antibody (Proteintech, 20543-1-AP) for IP. All co-IP experiments were performed in three independent biological replicates.

### 2.12 Multiplex Immunohistochemistry (mIHC)

A commercial tissue microarray (Bioaitech, F551101, Xi’an, China) containing 50 cases of TNBC tissues was used for protein expression analysis. Multiplex immunohistochemistry was performed using the Opal Multiplex IHC protocol (Akoya Biosciences, Marlborough, MA, USA). Briefly, TMA sections were deparaffinized and rehydrated through a series of xylene and graded alcohol washes. Antigen retrieval was performed using AR6 buffer (Akoya Biosciences, AR600) in a microwave. After blocking with 3% BSA in TBST for 1 h at room temperature, sections were incubated with primary antibodies against PARK2 (1:200, Proteintech, 66674-1-Ig) and PGK1 (1:200, Proteintech, 17811-1-AP) overnight at 4°C. The sections were then incubated with Opal fluorophore-conjugated secondary antibodies (1:100 dilution for both Opal 520, Akoya Biosciences, FP1487001KT; and Opal 570, Akoya Biosciences, FP1488001KT) for 10 min at room temperature, according to the manufacturer’s instructions. Nuclei were counterstained with DAPI (1 μg/mL, Thermo Fisher Scientific). Between each step, sections were washed three times with TBST. Fluorescent images were acquired using a Vectra Polaris Automated Quantitative Pathology Imaging System (Akoya Biosciences). The mean fluorescence intensity (MFI) for PARK2 and PGK1 was quantified using ImageJ software (version 1.54, National Institutes of Health). For quantification, five regions of interest (ROIs) encompassing representative tumor areas were randomly selected for each TMA core. All image analyses and MFI quantifications were performed by two independent investigators who were blinded to the clinical and pathological parameters of the samples.

### 2.13 Cell Migration and Invasion Assays

Cell migration and invasion were assessed using 24-well transwell chambers (8 μm pore size, Corning, 3422, Corning, NY, USA), using cells with indicated overexpression.

For migration assays, 5 × 10^4^ cells suspended in 200 μL of serum-free medium (DMEM, Procell, PM150210, or RPMI-1640, Procell) were seeded in the upper chamber, with 600 μL of complete medium containing 10% FBS (Procell, 164210) in the lower chamber as a chemoattractant.

For invasion assays, chambers were pre-coated with 50 μL of Matrigel (BD Biosciences, 356234, San Jose, CA, USA) diluted 1:8 in serum-free medium and incubated at 37°C for 2 h to allow for gel polymerization. Following polymerization, 1 × 10^5^ cells suspended in 200 μL of serum-free medium were seeded into the upper chamber. After 24 h, cells on the upper surface were removed with cotton swabs. Migrated or invaded cells on the lower surface were fixed with 4% paraformaldehyde, stained with 0.1% crystal violet, and counted in five random fields under an inverted light microscope (Olympus IX73, Olympus). Each experiment was performed in triplicate.

### 2.14 Animal Model and Lung Metastasis Assay

Animal studies were approved by the Biomedical Ethics Review Committee of West China Hospital, Sichuan University (approval No. 20251821). A total of 24 female BALB/c nude mice (5 weeks old, 18–20 g) were purchased from Vital River Laboratory Animal Technology (Beijing, China) and housed under specific pathogen-free conditions with a 12-h light/dark cycle and free access to food and water. Only healthy mice exhibiting normal baseline body weight and behavior were included in the study. Exclusion criteria were defined a priori as failed tail vein injection or the development of unrelated health issues prior to the experimental endpoint; however, no animals met the exclusion criteria, and all 24 mice were included in the final analysis.

The sample size was determined based on a power analysis using G*Power software. Assuming a large effect size (Cohen’s d = 1.8) derived from preliminary *in vitro* data, a significance level (α) of 0.05, and a power (1 − β) of 80%, a minimum of 6 mice per group was required to detect statistically significant differences in metastatic burden.

Mice were randomly divided into four groups (n = 6 per group) and allowed to acclimate for one week before experimentation. The four experimental groups consisted of mice injected with BT549 cells stably expressing: (1) empty vector control, (2) PGK1 overexpression (PGK1-OE), (3) PARK2 overexpression (PARK2-OE), or (4) co-expression of both (PGK1-OE + PARK2-OE). 2 × 10^6^ cells from each stable cell line were resuspended in 200 μL PBS and injected into the lateral tail vein using a 27-gauge needle.

To minimize allocation and execution bias, animal cages were coded by a third party, and the investigator performing the injections and routine husbandry was blinded to the group assignments. This experimental model specifically evaluates the capacity of circulating tumor cells to extravasate and colonize the lung parenchyma, rather than reflecting the complete metastatic cascade that includes primary tumor growth, local invasion, and intravasation under natural clonal selection pressure.

Mice were monitored daily for signs of distress and body weight was recorded weekly. Humane endpoints were defined as a body weight loss of >20%, severe lethargy, hunched posture, or labored breathing. In this study, all mice remained in good condition and reached the pre-determined endpoint of 8 weeks without requiring early euthanasia; thus, no comparisons of metastatic burden between different time points were necessary. Eight weeks post-injection, mice were euthanized by CO_2_ asphyxiation followed by cervical dislocation. Lungs were immediately harvested, photographed, and fixed in 10% neutral buffered formalin for 24 h for subsequent H&E and immunohistochemical staining. Metastatic nodules were identified macroscopically on the lung surface and subsequently confirmed by histological examination. The primary outcome measure was the metastatic burden, which was calculated as the total area of metastatic lesions relative to the total lung area. To minimize observer bias, the identification and quantification of metastatic burden were performed in a blinded manner by two independent investigators unaware of the group allocations.

### 2.15 Histological Analysis

Fixed lung tissues were processed through graded alcohols, cleared in xylene, and embedded in paraffin. Serial sections (5 μm thickness) were cut using a rotary microtome (RM2235, Leica Biosystems, Wetzlar, Germany) at three different levels (100 μm apart) to ensure comprehensive analysis of metastatic burden. Hematoxylin and eosin (H&E) staining was performed using a commercial H&E Stain Kit (Servicebio, G1005, Wuhan, China). Sections were deparaffinized, rehydrated, and stained with Harris hematoxylin for 5 min, followed by eosin Y for 2 min. Slides were dehydrated, cleared, and mounted with DPX mounting medium (Sigma-Aldrich, 06522). Images of H&E-stained sections were captured and analyzed using an Olympus BX53 microscope equipped with cellSens Standard software (version 1.18, Olympus).

### 2.16 Immunohistochemistry

For immunohistochemical analysis, 5-μm-thick lung sections were deparaffinized and subjected to heat-induced antigen retrieval in citrate buffer (pH 6.0) for 20 min. Endogenous peroxidase activity was blocked with 3% H_2_O_2_ for 10 min, followed by blocking with 5% normal goat serum (Beyotime, C0265) for 1 h at room temperature. Sections were incubated overnight at 4°C with primary antibodies: anti-PGK1 (1:200, Abcam, ab199438, Cambridge, UK), anti-PARK2 (1:150, CST, #4211), or anti-Vimentin (1:300, CST, #5741). After washing, sections were incubated with HRP-conjugated secondary antibodies (Goat Anti-Rabbit IgG H&L (HRP), 1:500, Abcam, ab6721; or Goat Anti-Mouse IgG H&L (HRP), 1:500, Abcam, ab6789) for 1 h at room temperature. Signal was visualized using DAB substrate kit (Vector Laboratories, #SK-4100, Newark, CA, USA) for 3 min at room temperature and counterstained with hematoxylin. Images were captured using an Olympus BX53 microscope equipped with a DP74 camera (Olympus, Tokyo, Japan). Semi-quantitative analysis of IHC staining in lung metastatic lesions was performed by two independent investigators blinded to group allocation. For each mouse, at least three representative metastatic lesions were selected from lung sections, and staining was evaluated based on both staining intensity (negative, weak, moderate, or strong) and the fraction of positive tumor cells (<25%, 25–75%, or >75%). These two parameters were combined to generate a final protein expression score as follows: not detected = 0, low = 1, medium = 2, and high = 3. The mean score of the three lesions was calculated for each mouse and used for statistical analysis.

### 2.17 Statistical Analysis

Statistical analysis was performed using GraphPad Prism (v10.1, GraphPad Software, Inc., San Diego, CA, USA). All data are presented as mean ± SD from at least three independent experiments. Statistical significance was determined using Student’s *t*-test for two-group comparisons or one-way ANOVA with Tukey’s post hoc test for multiple group comparisons. Correlation analyses were performed using Spearman’s rank correlation coefficient. Survival analyses were performed using the Kaplan-Meier method with log-rank test. *p*-values < 0.05 were considered statistically significant.

## 3 Results

### 3.1 PGK1 Expression Is Associated with Unfavorable Survival in Patients with TNBC

**Figure 1:**
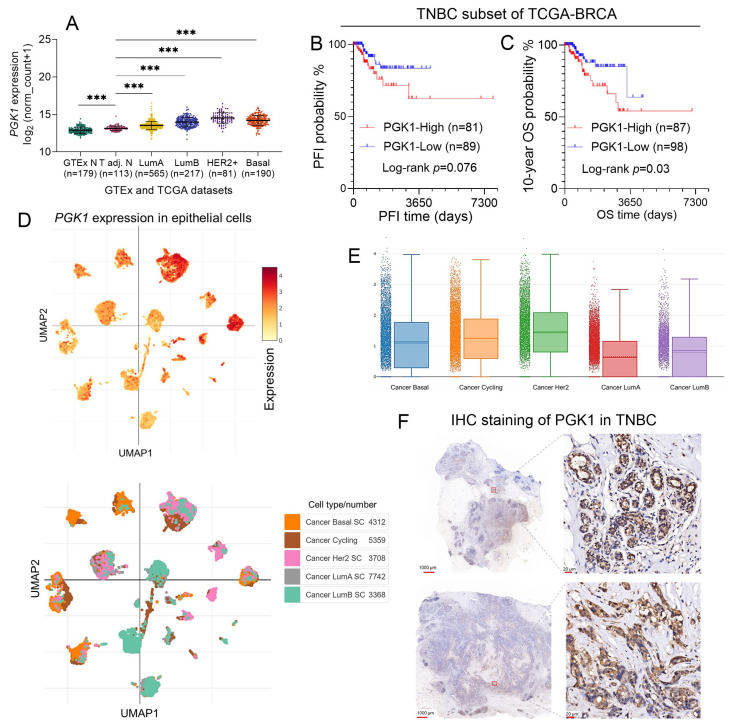
***Phosphoglycerate kinase 1* (*PGK1*) expression is associated with unfavorable survival in patients with triple-negative breast cancer (TNBC).** (**A**) Scatter plot showing gene expression of PGK1 across various breast cancer subtypes and normal tissues, as derived from the combined dataset of GTEx and TCGA. GTEx N: GTEx normal breast tissue; T adj.: TCGA normal adjacent tissue; LumA: luminal A; LumB: luminal B; Human Epidermal Growth Factor Receptor 2 (HER2)+: HER2-enriched; Basal: basal-like. (**B**) Progression-free interval (PFI) and (**C**) overall survival (OS) for patients in the TNBC subset of TCGA-BRCA cohort stratified by *PGK1* expression levels (high vs. low), as analyzed by the log-rank test. (**D**) UMAP visualizations showing PGK1 expression in epithelial cells from single-cell RNA-seq data, with different cell clusters colored based on *PGK1* expression levels (left) and cell types (right). Cell type color coding and cell counts are indicated in the legend. (**E**) Distribution of PGK1 expression in distinct major subtypes of epithelial breast cancer cells (Cancer Basal SC, Cancer Cycling, Cancer Her2 SC, Cancer Luma SC, Cancer Lumb SC). (**F**) Representative Immunohistochemistry (IHC) staining of PGK1 in TNBC tissue sections. ***p < 0.001.

**Figure A1:**
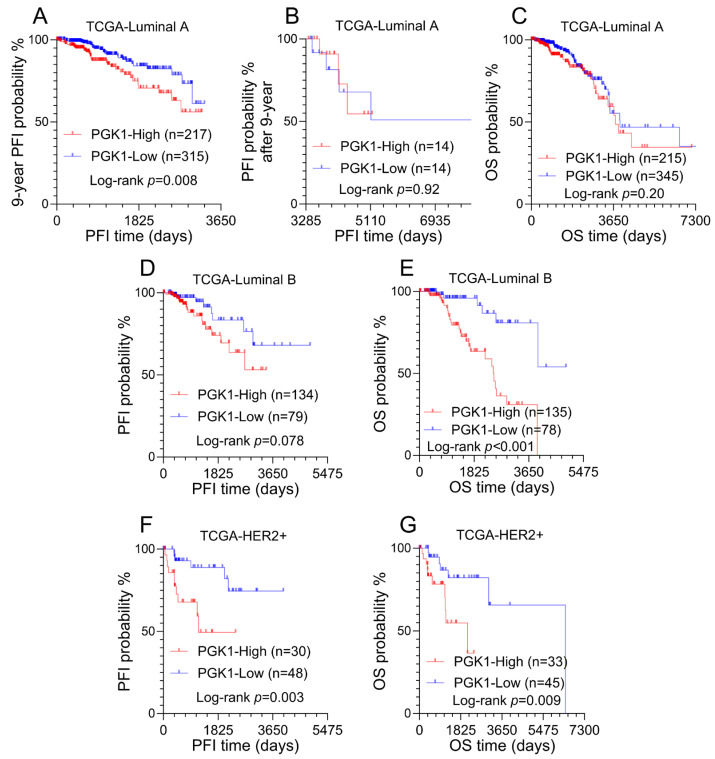
**Kaplan-Meier survival curves for progression-free interval (PFI) and overall survival (OS) stratified by phosphoglycerate kinase 1 (*PGK1*) expression in various breast cancer subtypes within the TCGA-BRCA cohort.** (**A**,**B**) PFI probability over 9 years (**A**) and after 9 years (**B**) for Luminal A subtype patients based on the optimal cutoff of *PGK1* expression. (**C**) OS probability for Luminal A subtype patients (PGK1-High, n = 215; PGK1-Low, n = 345), indicating no significant correlation with PGK1 expression (log-rank *p* = 0.20). (**D**,**E**) PFI (**D**) and OS (**E**) probability for Luminal B subtype patients. (**F**,**G**) PFI (**F**) and OS (**G**) probability for HER2^+^ subtype patients.

Using available data from TCGA and GTEx, it was observed that *PGK1* expression was significantly elevated in various breast cancer subtypes compared to normal tissues (Fig. 1A). Notably, within the TNBC subset of the TCGA-BRCA cohort, high *PGK1* expression was associated with a trend toward shorter PFI (log-rank *p* = 0.11) (Fig. 1B) and significantly reduced OS (HR: 2.138, 95%CI:1.001 to 4.569, log-rank *p* = 0.049) (Fig. 1C). PFI was selected as a key endpoint in this study because it serves as a more direct indicator of tumor aggressiveness and recurrence risk. This association also extended to some other subtypes (Fig. A1A–G). In the luminal A subtype, high *PGK1* expression significantly impacted 9-year PFI (log-rank *p* = 0.008) (Fig. A1A). In the luminal B subtype, high *PGK1* levels were associated with a significantly reduced OS (log-rank *p* < 0.001) (Fig. A1E). Similarly, for the HER2^+^ subtype, high *PGK1* expression was significantly correlated with shorter PFI (log-rank *p* = 0.003) and reduced OS (log-rank *p* = 0.009) (Fig. A1F,G).

Single-cell (SC) RNA-seq analysis revealed heterogeneous expression of *PGK1* across different epithelial cell clusters, with pronounced expression in certain clusters, particularly Basal and Cancer Cycling cells (Fig. 1D). Scatterplot comparison showed that *PGK1* expression varied among epithelial cancer cell subtypes, with Cancer Basal SC and Cancer HER2 SC displaying elevated levels compared to LumA and LumB SCs (Fig. 1E). IHC staining further confirmed PGK1 protein expression in TNBC tissues (Fig. 1F).

Given that PGK1 can be degraded via the ubiquitin-proteasomal pathway [17,23], potential ubiquitin E3 ligases regulating its degradation were explored using BioGRID (Table 1). Among the predicted E3 ligases, PARK2 can promote breast cancer cell apoptosis [27] and also acts as a tumor suppressor in multiple types of cancer [28,29]. NEDD4 helps maintain the cancer stem cell characteristics of breast cancer [30,31]. Consequently, these two E3 ligases were selected for further testing.

**Table 1:** Predicted E3 ligase interacted with Phosphoglycerate kinase 1 (PGK1) in BioGRID.

PGK1 Interactor	Organism	Description	Evidence
PARK2	H. sapiens	parkin RBR E3 ubiquitin protein ligase	3
NEDD4	H. sapiens	neural precursor cell expressed, developmentally down-regulated 4	1
UBR5	H. sapiens	ubiquitin protein ligase E3 component n-recognin 5	1
HUWE1	H. sapiens	HECT, UBA and WWE domain containing 1	1
UBE3A	H. sapiens	ubiquitin protein ligase E3A	1
TRIM72	H. sapiens	tripartite motif containing 72	1

Abb: PGK1, Phosphoglycerate kinase 1; PARK2, Parkinson disease 2 (Parkin); NEDD4, Neural precursor cell expressed, developmentally down-regulated 4; UBR5, Ubiquitin protein ligase E3 component n-recognin 5; HUWE1, HECT, UBA and WWE domain containing 1; UBE3A, Ubiquitin protein ligase E3A; TRIM72, Tripartite motif containing 72; RBR, RING-between-RING; HECT, Homologous to the E6-AP Carboxyl Terminus; UBA, Ubiquitin-associated; WWE, Tryptophan-Tryptophan-Glutamate.

### 3.2 PARK2 Negatively Regulates the Stability of PGK1 Protein in TNBC

**Figure 2:**
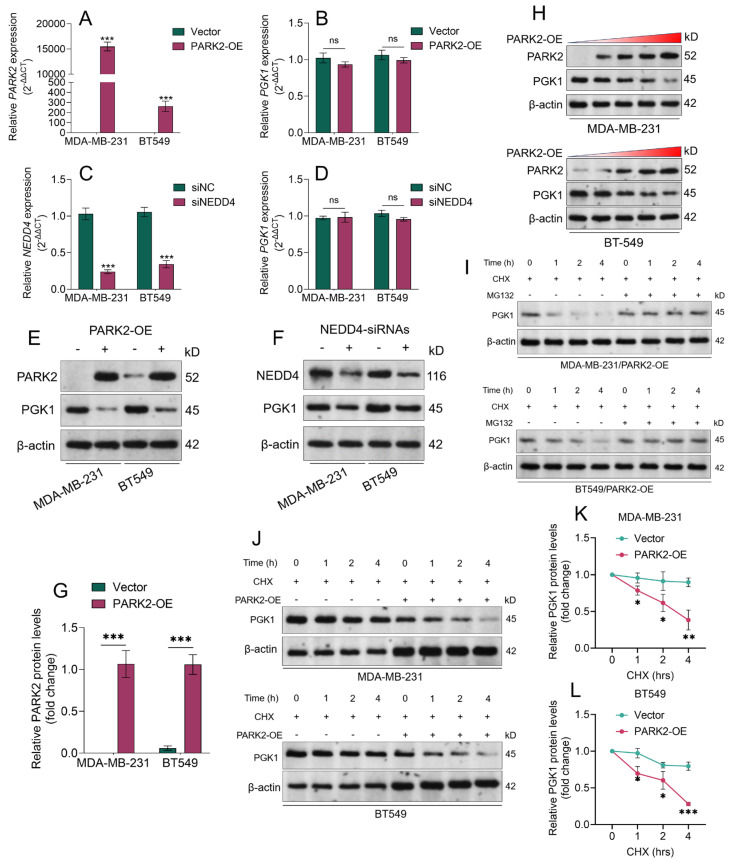
**Parkinson disease 2 (PARK2) negatively regulates the stability of PGK1 protein.** (**A**,**B**) Relative mRNA expression levels of *PARK2* (**A**) and *PGK1* (**B**) in MDA-MB-231 and BT-549 cells with *PARK2* overexpression (PARK2-OE). (**C**,**D**) Relative mRNA levels of *neural precursor cell expressed, developmentally down-regulated 4* (*NEDD4*) (**C**) and *PGK1* (**D**) in MDA-MB-231 and BT-549 cells with *NEDD4* knockdown (siNEDD4). (**E**) PARK2 and PGK1 protein levels in MDA-MB-231 and BT549 cells with or without *PARK2* overexpression. (**F**) NEDD4 and PGK1 protein levels in MDA-MB-231 and BT549 cells with or without *NEDD4* knockdown. (**G**) Quantification of relative PARK2 protein levels in MDA-MB-231 and BT549 cells after *PARK2* overexpression. (**H**) Dose-dependent overexpression of *PARK2* and corresponding alterations of PGK1 expression in MDA-MB-231 and BT-549 cells. (**I**) MDA-MB-231 and BT-549 cells *PARK2* overexpression were treated with cycloheximide (CHX, 50 μg/mL) and/or MG132 (5 μM) for indicated time. Then, western blotting analysis was performed to detect the cellular PGK1 levels. (**J**,**L**) Cycloheximide (CHX) chase assays were performed to assess PGK1 protein levels in MDA-MB-231 and BT-549 cells with or without *PARK2* overexpression (**J**). PGK1 protein levels in MDA-MB-231 (**K**) and BT-549 (**L**) cells at the indicated time points were quantified. *, *p* < 0.05; **, *p* < 0.01; ***, *p* < 0.001, ns: not significant.

To investigate the regulatory effects of PARK2 and NEDD4 on PGK1 protein stability, MDA-MB-231 and BT-549 cells were subjected to *PARK2* overexpression (PARK2-OE) and *NEDD4* (siNEDD4) knockdown. The mRNA levels of *PGK1* remained unchanged upon *PARK2* overexpression or *NEDD4* knockdown, indicating that PARK2 and NEDD4 do not affect *PGK1* transcription (Fig. 2A–D). *PARK2* overexpression significantly reduced PGK1 protein levels in both cell lines (Fig. 2E), whereas *NEDD4* knockdown did not alter PGK1 protein levels (Fig. 2F), confirming that NEDD4 does not directly regulate PGK1 under the experimental conditions. Quantification further showed a significant increase in PARK2 protein levels after PARK2 overexpression in both MDA-MB-231 and BT549 cells (Fig. 2G).

In a dose-dependent study, increasing *PARK2* expression led to a progressive reduction in PGK1 protein levels (Fig. 2H), suggesting a negative regulatory effect of PARK2 on PGK1 protein stability. To validate this, cells overexpressing PARK2 were treated with cycloheximide (CHX), a protein synthesis inhibitor. Western blotting revealed that PARK2 overexpression accelerated PGK1 protein degradation, and this effect was attenuated by the proteasome inhibitor MG132, indicating that PARK2 promotes proteasomal degradation of PGK1 (Fig. 2I). CHX chase assays further confirmed that PARK2 overexpression accelerates PGK1 protein turnover (Fig. 2J–L). Collectively, these results suggest that PARK2 negatively regulates the stability of PGK1 protein via proteasomal degradation pathways in MDA-MB-231 and BT-549 cells.

### 3.3 PARK2 Protein Expression Is Negatively Correlated with PGK1 Protein Expression in TNBC

**Figure 3:**
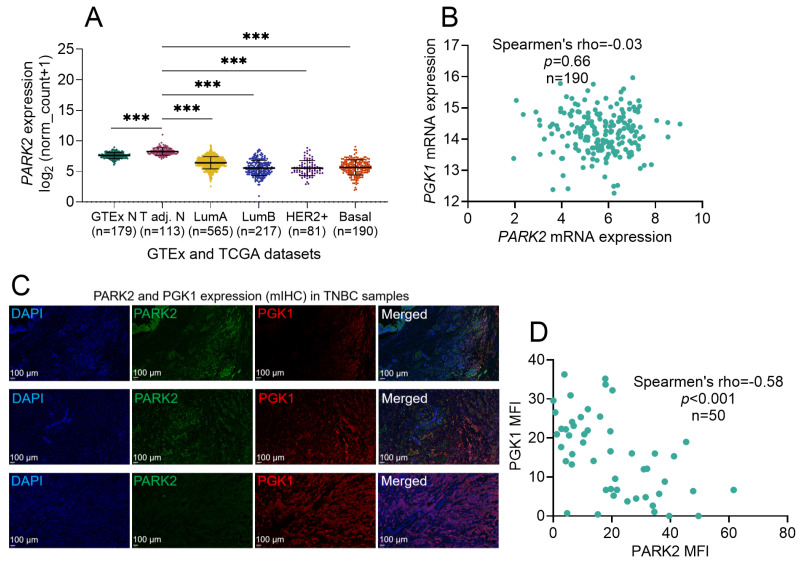
**PARK2 protein, but not mRNA, expression is negatively correlated with PGK1 protein expression in TNBC.** (**A**) PARK2 mRNA expression levels across normal breast tissue and different breast cancer subtypes, as analyzed from GTEx and TCGA datasets. (**B**) Correlation analysis of *PARK2* and *PGK1* mRNA expression in basal-like breast cancer samples from TCGA, showing no significant association (*p* = 0.66). (**C**) Representative multiplex immunohistochemistry (mIHC) images showing the expression and spatial distribution of PARK2 (green) and PGK1 (red) in TNBC tissue samples, with DAPI (blue) marking nuclei. (**D**) Quantitative analysis of protein expression in 50 TNBC samples reveals a significant negative correlation between PARK2 and PGK1 protein levels (Spearman’s rho = −0.58, *p* < 0.001). Scale bars, 100 μm. ***p < 0.001.

Analysis of TCGA and GTEx datasets revealed that *PARK2* mRNA expression was significantly lower in various breast cancer subtypes compared to normal breast tissue, with particularly reduced levels in basal-like breast cancers (Fig. 3A). In basal-like breast cancer samples from TCGA, no significant association was observed between *PARK2* and *PGK1* at the mRNA level (Spearman’s rho = −0.03, *p* = 0.66, n = 190) (Fig. 3B). This observation is consistent with the *in vitro* data showing that PARK2 regulates PGK1 at the post-transcriptional level rather than affecting its mRNA expression.

Multiplex immunohistochemistry (mIHC) on 50 TNBC tissue specimens was performed to investigate the relationship between these proteins in tumor samples. Representative images show the spatial distribution of PARK2 (green) and PGK1 (red) within tumor tissues (Fig. 3C). Interestingly, co-localization was observed between these proteins, suggesting potential physical interaction *in vivo*. Quantitative analysis revealed a significant negative correlation between PARK2 and PGK1 protein levels (Spearman’s rho = −0.58, *p* < 0.001) (Fig. 3D).

### 3.4 PARK2 Interacts with PGK1 in TNBC Cells

**Figure 4:**
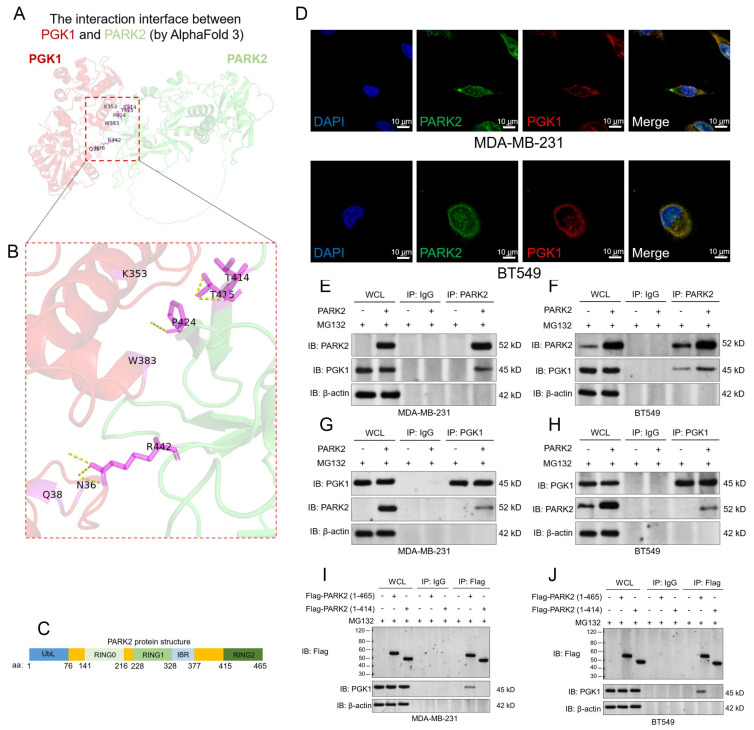
**PARK2 interacts with PGK1 in TNBC cells.** (**A**) Structural model of the predicted interaction between PGK1 (red) and PARK2 (green) by AlphaFold 3. (**B**) The inset displays the residues involved in the interaction. (**C**) Schematic illustration of PARK2 protein structure. (**D**) Immunofluorescence staining demonstrating the co-localization of PARK2 and PGK1 in MDA-MB-231 and BT549 TNBC cell lines. DAPI (blue) was used to stain nuclei, PARK2 is stained green, and PGK1 is stained red. The merged images indicate co-localization (yellow). Scale bars represent 10 μm. (**E**,**F**) Co-IP assays showing interaction between PARK2 and PGK1 in TNBC cell lines. Whole cell lysates (WCL) from MDA-MB-231 (**E**) and BT549 (**F**) were subjected to immunoprecipitation with an anti-PARK2 antibody and immunoblotted with anti-PARK2 and anti-PGK1 antibodies. MG132 treatment was used to inhibit proteasomal degradation pathways. WCL from both cell lines was also blotted as a control. (**G**,**H**) Co-IP assays showing reciprocal immunoprecipitation with an anti-PGK1 antibody, confirming the interaction with PARK2 in MDA-MB-231 (**G**) and BT549 (**H**) cell lines. (**I**,**J**) Co-IP assays in MDA-MB-231 (**I**) and BT549 (**J**) cell lines using Flag-tagged PARK2 mutants (aa 1-414 and aa 1-465). Whole cell lysates and immunoprecipitates by anti-Flag antibody were subjected to the detection of Flag-tagged proteins and PGK1.

To characterize the interaction between PARK2 and PGK1 in TNBC cells, AlphaFold 3 was used to illustrate the structural interface between these proteins (Fig. 4A). The specific interface between PARK2 (depicted in green) and PGK1 (depicted in red) highlighted multiple residues on PARK2 in the RING2 domain, including T414, T415, P442 and R442 (Fig. 4B,C). It’s known that the RING1 domain is essential for E2 enzyme binding, while the RING2 domain is critical for its substrate binding [26,32].

Immunofluorescence staining was conducted to investigate the spatial relationship between PARK2 and PGK1 in TNBC cell lines. In both MDA-MB-231 and BT549 cells, significant co-localization of PARK2 and PGK1 (yellow fluorescence) was observed, indicating that these proteins are in close proximity within the cells (Fig. 4D).

Co-IP assays were performed to confirm the physical interaction between PARK2 and PGK1 in MDA-MB-231 and BT549 cell lines. WCLs were immunoprecipitated with an anti-PARK2 antibody and subsequently immunoblotted using antibodies against PARK2 and PGK1. PARK2 was successfully immunoprecipitated in both cell lines, and PGK1 was detected in the precipitates, confirming their interaction (Fig. 4E,F). Reciprocal Co-IP assays using an anti-PGK1 antibody further validated the interaction in these TNBC cell lines (Fig. 4G,H).

To reveal the region of PARK2 responsible for interaction with PGK1, Flag-tagged PARK2 deletion mutant (aa 1-414) and the full-length counterpart (aa 1-465) were generated. Co-IP assays were conducted using MDA-MB-231 (Fig. 4I) and BT549 (Fig. 4J) cell lines with overexpression of the indicated mutants. Specifically, the PARK2 aa 1-414 deletion mutant lost the capability to bind PGK1, confirming the importance of the RING2 domain for this interaction.

### 3.5 PARK2 Induces K48 Polyubiquitination of PGK1

**Figure 5:**
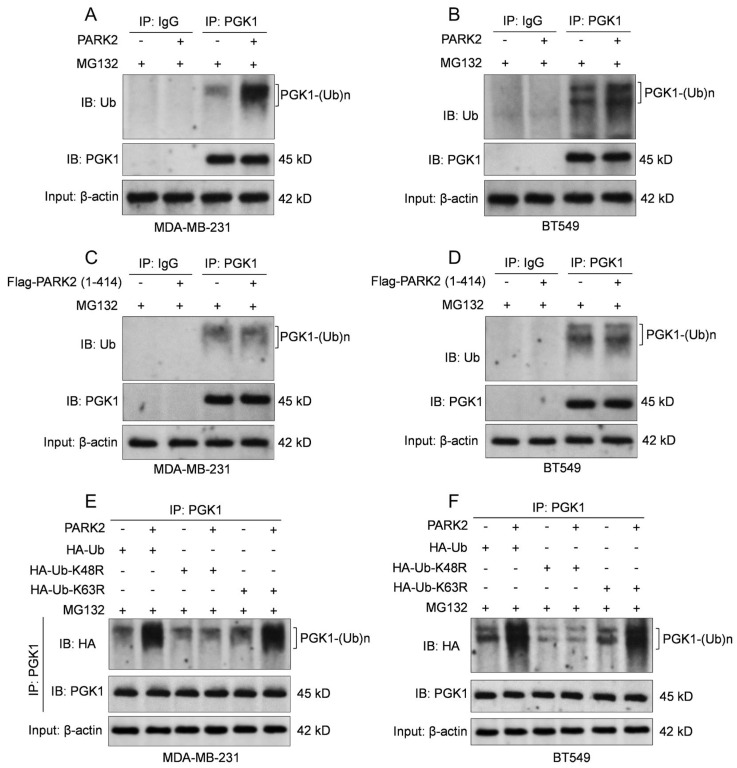
**PARK2 induces K48 polyubiquitination of PGK1.** (**A**,**B**) Cells were transfected with either control or *PARK2* expression vectors and treated with the proteasome inhibitor MG132 to prevent degradation of ubiquitinated proteins. WCL from MDA-MB-231 (**A**) and BT549 (**B**) were subjected to immunoprecipitation (IP) using an anti-PGK1 antibody, followed by immunoblotting (IB) with anti-ubiquitin (Ub) and anti-PGK1 antibodies. (**C**,**D**) Cells were transfected with either Flag-tagged full-length PARK2 or Flag-PARK2 (1-414) deletion mutant and treated with MG132. WCL from MDA-MB-231 (**C**) and BT549 (**D**) were immunoprecipitated with an anti-PGK1 antibody, followed by IB with anti-ubiquitin (Ub) and anti-PGK1 antibodies. (**E**,**F**) Cells were co-transfected with PARK2 and either HA-tagged wild-type ubiquitin (HA-Ub), K48R ubiquitin mutant (HA-Ub-K48R), or K63R ubiquitin mutant (HA-Ub-K63R) constructs, and treated with MG132. WCL from MDA-MB-231 (**E**) and BT549 (**F**) were immunoprecipitated using an anti-PGK1 antibody, followed by IB with anti-HA and anti-PGK1 antibodies. PGK1-(Ub)n indicates polyubiquitinated PGK1.

To determine whether PARK2 mediates polyubiquitination of PGK1, co-IP and immunoblotting (IB) experiments were performed in MDA-MB-231 and BT549 cells. Cells were treated with the proteasome inhibitor MG132 to prevent the degradation of ubiquitinated proteins, allowing the accumulation and detection of ubiquitinated PGK1. Overexpression of *PARK2* enhanced polyubiquitination of PGK1 compared to the control in both cell lines (Fig. 5A,B). However, the PARK2 deletion mutant (aa 1-414) failed to enhance the ubiquitination of PGK1 (Fig. 5C,D). This result underscores the importance of the RING2 domain in PARK2 for exerting its E3 ligase activity to catalyze PGK1 ubiquitination.

To ascertain the specific type of ubiquitin linkage involved, cells were co-transfected with HA-tagged wild-type ubiquitin (HA-Ub), K48R ubiquitin mutant (HA-Ub-K48R), or K63R ubiquitin mutant (HA-Ub-K63R) constructs. Co-IP assays in both MDA-MB-231 and BT549 cells revealed substantial ubiquitination of PGK1 in cells expressing wild-type ubiquitin, which was significantly reduced in cells expressing the HA-Ub-K48R mutant (Fig. 5E,F). In contrast, ubiquitination was largely unaffected in cells expressing the HA-Ub-K63R mutant. These findings indicate that PARK2 predominantly mediates K48-linked polyubiquitination of PGK1, marking it for proteasomal degradation in TNBC cells.

### 3.6 PARK2 Reduces Mesenchymal Phenotypes of Breast Cancer Cells via PGK1

**Figure 6:**
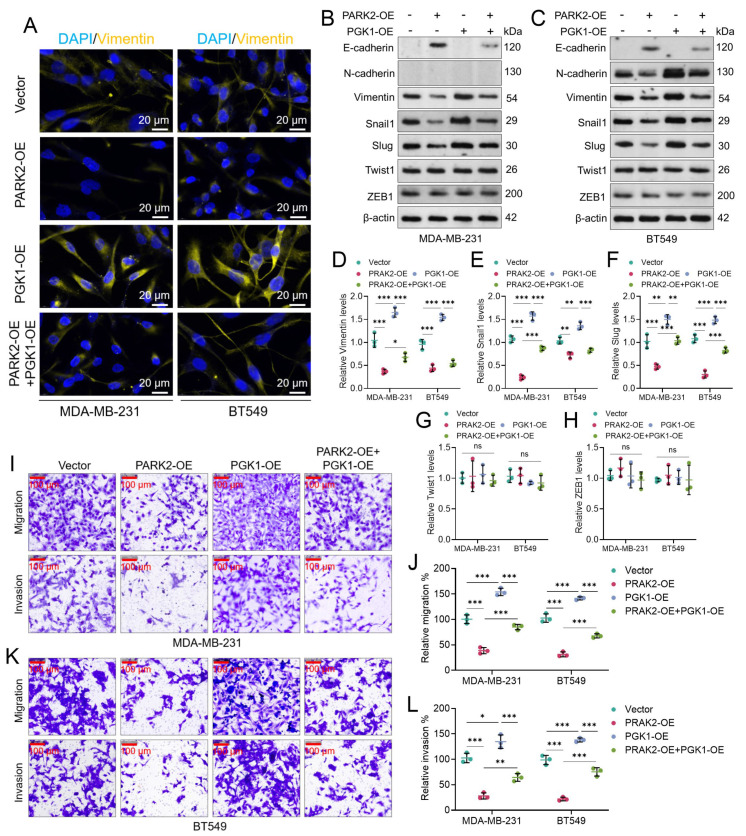
**PARK2 reduces mesenchymal phenotypes of breast cancer cells via PGK1.** (**A**) Immunofluorescence staining of Vimentin (yellow) in MDA-MB-231 and BT549 cells transfected with vector control, PARK2 overexpression (PARK2-OE), PGK1 overexpression (PGK1-OE), or both PARK2-OE and PGK1-OE. Scale bars, 20 μm. (**B**,**C**) Western blot analysis of epithelial-mesenchymal transition (EMT) markers (E-cadherin, N-cadherin, Vimentin) and EMT-associated transcription factors (Snail1, Slug, Twist1, ZEB1) in MDA-MB-231 (**B**) and BT549 (**C**) cells under the indicated conditions. (**D**–**H**) Quantification of relative protein levels of Vimentin (**D**), Snail1 (**E**), Slug (**F**), Twist1 (**G**) and ZEB1 (**H**) in MDA-MB-231 and BT549 cells, as determined by densitometric analysis of western blots. (**I**–**L**) Transwell migration and invasion assays in MDA-MB-231 (**I**,**J**) and BT549 (**K**,**L**) cells under the indicated conditions, with representative images shown. Quantification of relative migration (**J**) and invasion (**L**) percentages are presented for each group. *, *p* < 0.05; **, *p* < 0.01; ***, *p* < 0.001, ns, no significance.

**Figure A2:**
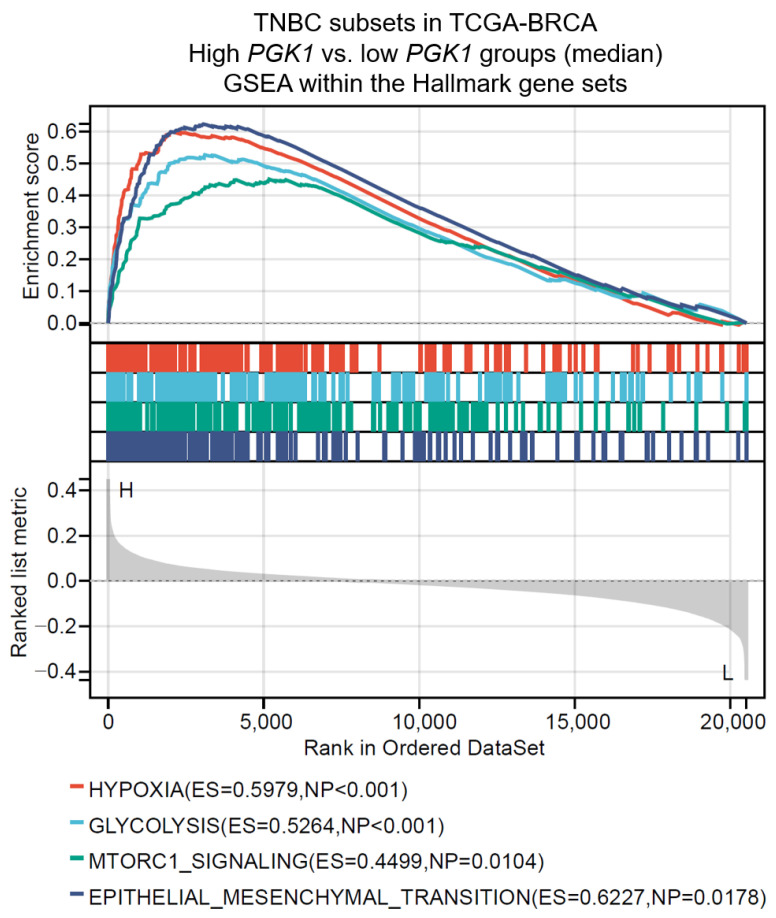
**High *PGK1* expression is associated with enrichment of epithelial-to-mesenchymal transition (EMT) and related pathways in triple-negative breast cancer (TNBC).** Gene set enrichment analysis (GSEA) of TCGA-BRCA TNBC subsets. Comparison was performed between high *PGK1* and low *PGK1* expression groups (stratified by median). Enrichment plots are shown for selected Hallmark gene sets, including HYPOXIA (enrichment score (ES) = 0.5979, Nominal *p*-value (NP) < 0.001), GLYCOLYSIS (ES = 0.5264, NP < 0.001), MTORC1 SIGNALING (ES = 0.4499, NP = 0.0104), and EPITHELIAL-MESENCHYMAL TRANSITION (EMT) (ES = 0.6227, NP = 0.0178). The ranked list metric and enrichment scores indicate that high PGK1 expression is significantly associated with upregulation of EMT and other aggressive tumor-related pathways in TNBC.

GSEA analysis showed a strong association between high *PGK1* expression and enrichment of EMT-related gene signatures in TNBC (Fig. A2). Immunofluorescence staining revealed that overexpression of *PARK2* (PARK2-OE) markedly reduced Vimentin expression in both MDA-MB-231 and BT549 cells, whereas *PGK1* overexpression (PGK1-OE) increased Vimentin levels (Fig. 6A). Notably, co-expression of PARK2 with PGK1 (PARK2-OE + PGK1-OE) partially reversed the pro-mesenchymal effect of PGK1 on Vimentin expression (Fig. 6A). Western blotting data showed that co-expression of PARK2 and PGK1 (PARK2-OE + PGK1-OE) resulted in significantly lower levels of Vimentin, Snail1, and Slug compared to PGK1-OE alone, but these levels remained higher than in PARK2-OE or vector controls (Fig. 6B–F), demonstrating that PARK2 only partially reverses PGK1-induced EMT.

N-cadherin was undetectable in MDA-MB-231 cells. However, in BT549 cells, PGK1-mediated elevation of N-cadherin was partially reversed by PARK2 overexpression (Fig. 6B,C). Twist1 and ZEB1 levels were not significantly altered among the groups (Fig. 6G,H).

Quantitative analysis of transwell migration and invasion assays revealed that co-expression of PARK2 and PGK1 significantly reduced migration and invasion compared to PGK1-OE alone, but did not fully restore these properties to the levels observed in vector or PARK2-OE cells (Fig. 6I–L), further supporting a partial rescue effect.

### 3.7 PARK2 Suppresses PGK1-Mediated Lung Metastasis In Vivo

**Figure 7:**
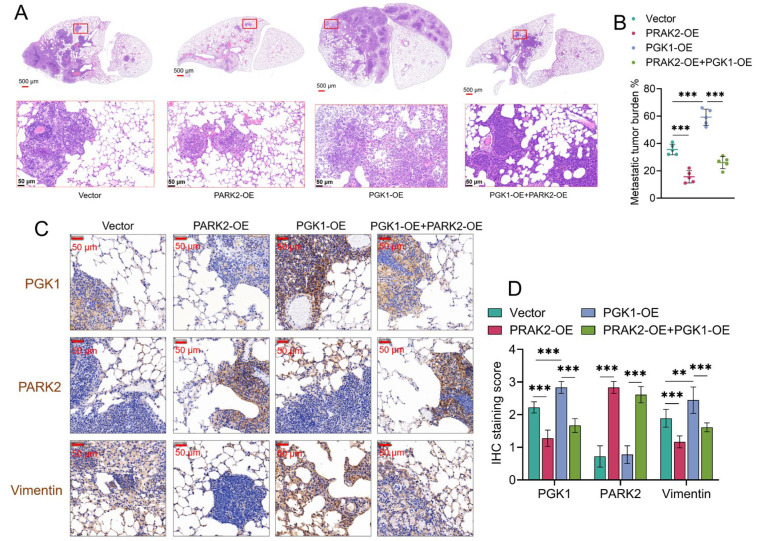
**PARK2 suppresses PGK1-driven lung metastasis and mesenchymal phenotype *in vivo*.** (**A**) Representative H&E-stained lung sections and enlarged areas from BALB/c nude mice injected via tail vein with BT549 cells stably expressing vector control, PGK1 overexpression (PGK1-OE), PARK2 overexpression (PARK2-OE), or co-expression of PGK1 and PARK2 (PGK1-OE + PARK2-OE). Scale bars: 500 μm for low-magnification images, 50 μm for high-magnification images. (**B**) Quantification of metastatic tumor burden in the lungs, expressed as the percentage of metastatic area relative to total lung area for each group (n = 6 mice per group). (**C**) Representative immunohistochemical staining of lung sections for PGK1, PARK2, and Vimentin in each experimental group. Scale bar, 50 μm. (**D**) Semi-quantitative analysis of IHC staining scores for PGK1, PARK2, and Vimentin in lung metastatic lesions (n = 6 mice per group). Data are presented as mean ± SD. **p < 0.01, ***p < 0.001 by one-way ANOVA with Tukey’s post hoc test.

To validate the functional significance of the PARK2-PGK1 regulatory axis *in vivo*, a lung metastasis model was generated using tail vein injection of BT549 cells. Mice were injected with BT549 cells stably expressing control vector, PGK1 overexpression (PGK1-OE), PARK2 overexpression (PARK2-OE), or co-expression of both proteins (PGK1-OE + PARK2-OE). After 8 weeks, histological examination of lung tissues revealed marked differences in metastatic burden among the experimental groups (Fig. 7A,B).

H&E staining demonstrated that PGK1 overexpression significantly promoted lung metastasis formation, with numerous large metastatic nodules distributed throughout the lung parenchyma. In contrast, PARK2 overexpression resulted in a dramatic reduction in both the number and size of metastatic lesions compared to the control group. Notably, co-expression of PARK2 with PGK1 substantially attenuated the pro-metastatic effects of PGK1, resulting in an intermediate phenotype between PGK1-OE alone and control groups (Fig. 7A). Quantitative analysis of metastatic burden confirmed these observations (Fig. 7B).

To investigate the molecular mechanisms underlying these phenotypic differences, immunohistochemical analysis of the lung sections were conducted. As expected, PGK1 staining intensity was markedly elevated in the PGK1-OE group, while PARK2-OE tumors showed minimal PGK1 expression (Fig. 7C,D), consistent with PARK2-mediated degradation of PGK1. In the co-expression group, PGK1 levels were intermediate, confirming that PARK2 can reduce PGK1 protein levels even when PGK1 is overexpressed (Fig. 7C,D).

Consistent with *in vitro* findings linking PGK1 to EMT regulation, Vimentin expression patterns closely mirrored PGK1 levels across all groups. Lung metastases from PGK1-OE mice exhibited strong Vimentin staining, indicative of enhanced mesenchymal characteristics. PARK2 IHC staining confirmed successful overexpression in the PARK2-OE and PGK1-OE + PARK2-OE groups, with nuclear and cytoplasmic localization patterns consistent with its known subcellular distribution (Fig. 7C,D). *PARK2* overexpression resulted in minimal Vimentin expression, while the co-expression group showed intermediate staining intensity. The correlation between PGK1 and Vimentin expression in metastatic lesions further supports the role of the PARK2-PGK1 axis in regulating partial EMT phenotypes during metastatic progression (Fig. 7C,D).

### 3.8 Validation of the PARK2-PGK1 Gene Expression Axis and Clinical Outcomes in TNBC Patients

**Figure A3:**
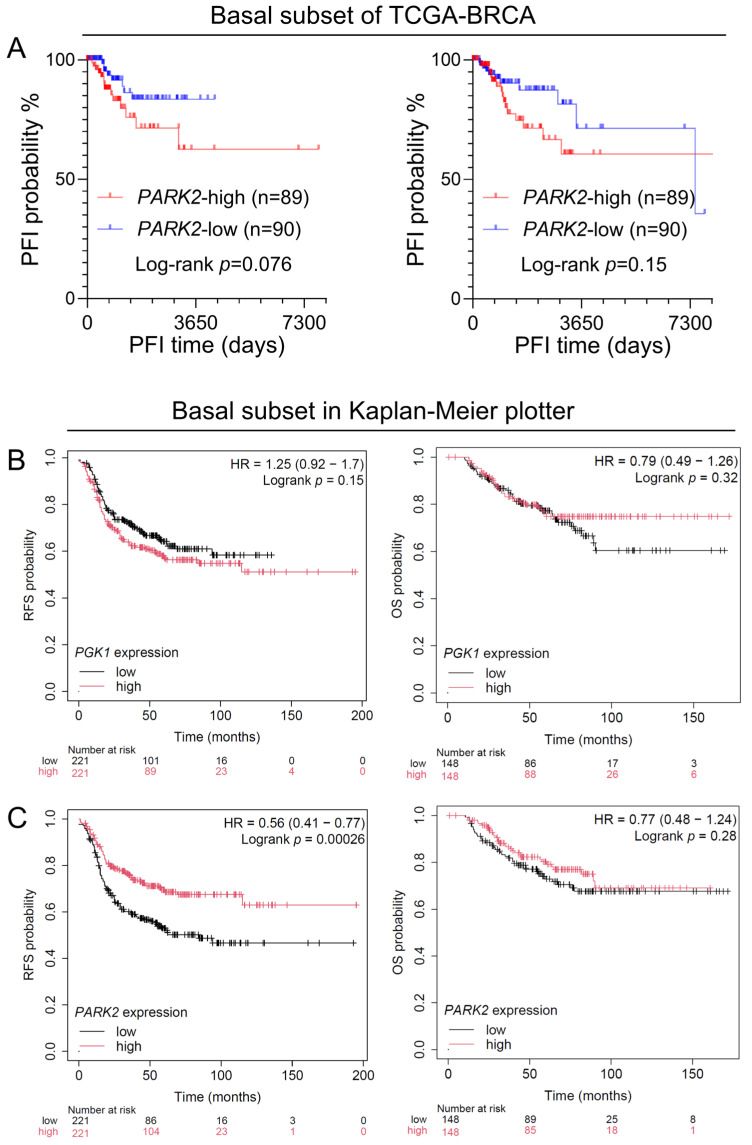
**Prognostic significance of *PARK2* and *PGK1* expression in basal-like breast cancer.** (**A**) Kaplan-Meier survival curves for progression-free interval (PFI) in the basal subset of TCGA-BRCA, stratified by PARK2 expression (**left**: log-rank *p* = 0.076; **right**: log-rank *p* = 0.15). (**B**) Kaplan-Meier plots from the Kaplan-Meier Plotter database showing relapse-free survival (RFS, **left**) and overall survival (OS, **right**) in basal-like breast cancer patients stratified by *PGK1* expression. (**C**) Kaplan-Meier plots for RFS (**left**) and OS (**right**) in basal-like breast cancer patients stratified by PARK2 expression. Hazard ratios (HR) and log-rank *p*-values are indicated.

**Figure A4:**
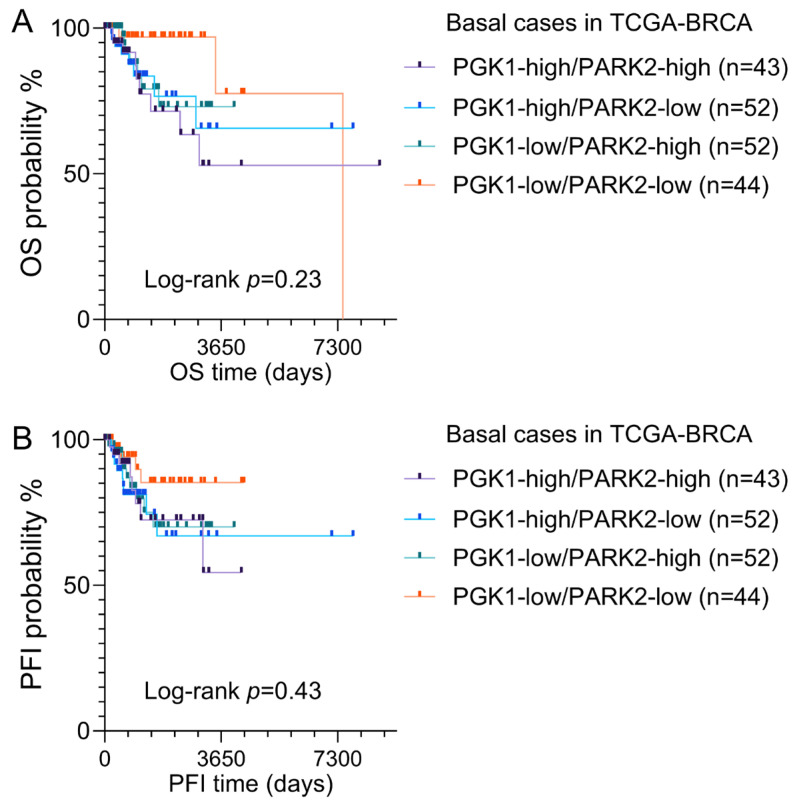
**Combined prognostic value of *PGK1* and *PARK2* expression in basal-like breast cancer.** (**A**) Kaplan-Meier survival curves for overall survival (OS) in basal-like breast cancer cases from TCGA-BRCA, stratified by combined median *PGK1* and *PARK2* expression levels (PGK1-high/PARK2-high, PGK1-high/PARK2-low, PGK1-low/PARK2-high, PGK1-low/PARK2-low). (**B**) Kaplan-Meier survival curves for progression-free interval (PFI) in the same patient groups as in (**A**). Log-rank *p*-values are indicated.

To further validate the clinical relevance of *PGK1* and *PARK2* in TNBC, additional survival analyses were performed. In the basal-like/TNBC subset of TCGA-BRCA, the prognostic value of *PARK2* expression in PFI (log-rank *p* = 0.076) and OS (log-rank *p* = 0.15) is limited (Fig. A3A). In comparison, the association between *PGK1* expression and survival outcomes, including recurrence-free survival (RFS) and OS were not confirmed in the Kaplan-Meier Plotter dataset (Fig. A3A,B). However, high *PARK2* expression was significantly associated with improved RFS (HR = 0.56, *p* = 0.00026) in the same dataset (Fig. A3C). To address the prognostic significance of combined PGK1/PARK2 expression, survival analysis was performed in the basal-like/TNBC subset of TCGA-BRCA. However, the combined PGK1-high/PARK2-low signature did not show a statistically significant association with OS or PFI (Fig. A3A,B). To address the prognostic significance of combined *PGK1*/*PARK2* expression, patients in the TCGA-BRCA basal-like subset were divided into four groups based on median expression of both genes. While it was hypothesized that the *PGK1*-high/*PARK2*-low group would exhibit the worst outcomes, the combined signature did not show a statistically significant association with OS or PFI at the mRNA level (Fig. A4A,B). These results suggest that while PARK2 and PGK1 protein levels are strongly coupled via post-translational regulation, their mRNA levels—often used in large-scale genomic databases—may not fully capture the functional activity of the PARK2-PGK1 axis.

## 4 Discussion

TNBC remains one of the most aggressive and therapeutically challenging subtypes of breast cancer, largely due to its lack of hormone receptors and HER2 expression, which precludes the use of targeted therapies [33]. The identification of novel molecular vulnerabilities in TNBC is of paramount importance [34]. This study provided compelling evidence that the E3 ubiquitin ligase PARK2 acts as a negative regulator of PGK1 protein stability in TNBC, thereby suppressing epithelial-mesenchymal transition (EMT), cell invasion, and metastasis. Findings of this study not only elucidate a previously unrecognized post-translational regulatory mechanism of PGK1 in breast cancer but also highlight the therapeutic potential of targeting the PARK2-PGK1 axis in TNBC.

Our analysis of TCGA and GTEx datasets confirmed that *PGK1* is significantly upregulated across all breast cancer subtypes, with high expression correlating with poor patient outcomes. This prognostic association was particularly prominent in TNBC, where elevated PGK1 levels significantly reduced overall survival [35,36]. This upregulation is likely driven by a complex interplay between metabolic reprogramming and epigenetic modifications. Recent evidence suggests a positive feedback loop in breast cancer where glycolysis-derived lactate triggers histone lactylation (specifically H4K79la and H4K91la), which in turn epigenetically activates the transcription of glycolytic genes, including PGK1 [37]. Single-cell RNA-seq analysis further revealed heterogeneous PGK1 expression patterns within tumor epithelial cells, with particularly high levels in basal and cycling cancer cells, populations known to drive tumor aggressiveness and therapy resistance. These findings establish PGK1 as a clinically relevant target in TNBC and provide the rationale for investigating its post-translational regulation.

The oncogenic functions of PGK1 in breast cancer extend well beyond its canonical role in glycolysis, making it an attractive therapeutic target. Previous studies have shown that elevated PGK1 levels promote the formation of breast cancer stem cells through metabolic reprogramming. Elevated PGK1 levels catalyze the conversion of 1,3-bisphosphoglycerate (1,3-BPG) to 3-phosphoglycerate (3-PG), leading to the accumulation of 3-PG and increased serine synthesis, accumulation of S-adenosylmethionine (SAM), and trimethylation of histone H3 at lysine 4 (H3K4me3). Activation of H3K4me3 promotes the formation of breast cancer stem cells (BCSCs) by increasing the transcriptional levels of stem cell-related factors [20]. PGK1 also participates in non-metabolic functions, including EGFR trafficking [38] and DNA replication through its interaction with CDC7 kinase [39]. The expression of EGFR in breast cancer cells is closely related to MAPK signaling activation and ERα transcription [40,41]. Recent studies have highlighted how diverse post-translational modifications (PTMs) fine-tune PGK1 activity to drive malignancy. For instance, endogenous hydrogen sulfide (H_2_S) mediates PGK1 persulfidation at Cys108 and Cys316, significantly enhancing its glycolytic activity and promoting breast cancer metastasis [42]. Furthermore, in TNBC, the enzyme OXCT1 has been shown to increase PGK1 protein stability by promoting its succinylation at K146, which physically hinders ubiquitination and promotes immune escape [43]. These diverse functions underscore PGK1’s role as a multifunctional protein that integrates metabolic and signaling pathways to promote tumorigenesis.

A central discovery of this study is the identification of PARK2 as a critical negative regulator of PGK1 stability through ubiquitin-mediated proteasomal degradation. While PARK2 is traditionally known for its role in mitophagy [44], findings of this study align with an emerging paradigm where PARK2 acts as a versatile tumor suppressor by targeting various non-mitochondrial oncogenic proteins. In clear cell renal cell carcinoma, PARK2 has been shown to target CKS2 for degradation to decrease tumor aggressiveness [45]. Similarly, in hepatocellular carcinoma, PARK2 modulates the immune microenvironment by inducing the ubiquitination and degradation of PD-1 [46]. Interestingly, the regulation of PARK2 itself is becoming clearer; for example, in gastric cancer, a novel circRNA-encoded protein (SEMA3C-319aa) has been found to enhance PARK2 transcription to promote GPX4 degradation and ferroptosis [47]. PARK2 physically interacts with PGK1, with the RING2 domain of PARK2 being essential for this interaction and subsequent E3 ligase activity. This direct interaction is a vulnerable node in cancer cells. For instance, in colon cancer, Fibronectin 1 (FN1) has been shown to competitively inhibit the interaction between PARK2 and its substrates, thereby stabilizing oncogenic proteins [48].

Functionally, the PARK2-PGK1 axis was shown to play a pivotal role in regulating metastatic phenotypes in TNBC cells. *PARK2* overexpression suppressed migration and invasion, whereas *PGK1* overexpression promoted these phenotypes. Importantly, PARK2 counteracted the pro-invasive and pro-migratory effects of PGK1. At the molecular level, these functional changes were accompanied by selective alterations in EMT-associated markers, including Vimentin, Snail1, Slug, and E-cadherin, whereas other canonical EMT markers showed relatively limited or inconsistent changes. In addition, since the *in vivo* analysis included Vimentin as a mesenchymal marker but did not incorporate a broader panel such as E-cadherin and N-cadherin, the *in vivo* data should be interpreted as supporting selective mesenchymal-marker modulation rather than full EMT reprogramming. Future studies including expanded *in vivo* marker panels will further clarify the extent to which the PARK2-PGK1 axis influences epithelial-mesenchymal plasticity during metastasis. Therefore, these data reflect partial EMT-associated remodeling, or EMT-linked metastatic plasticity, rather than a complete canonical EMT program.

While this study focused on PARK2, it is noteworthy that PGK1 stability is regulated by multiple E3 ligases and deubiquitinases, suggesting a complex regulatory network. For instance, KIF15 facilitates PGK1 degradation by scaffolding its interaction with USP10 [23], while STUB1 promotes PGK1 ubiquitination following interaction with LINC00926 [17]. Conversely, certain long non-coding RNAs such as SNHG26, NRSN2-AS1 and circSTT3A can stabilize PGK1 by inhibiting its ubiquitination [20,49,50]. This regulatory complexity suggests that PGK1 levels are tightly controlled through multiple mechanisms, and disruption of any component could contribute to tumorigenesis.

A notable observation in this study is the discrepancy between the prognostic value of *PARK2/PGK1* mRNA levels and the functional/protein-level data. While mIHC analysis of TNBC tissues demonstrated a significant negative correlation between PARK2 and PGK1 protein levels (r = −0.58), survival analysis based on mRNA expression in TCGA and Kaplan-Meier Plotter yielded mixed results. Specifically, while PARK2 mRNA levels were significantly associated with better RFS in some cohorts, the combined mRNA signature of PGK1 and PARK2 failed to reach statistical significance for overall survival. This decoupling between mRNA levels and clinical outcomes is likely explained by the mechanistic finding that PARK2 regulates PGK1 primarily at the post-translational level. Because PARK2 induces the degradation of PGK1 protein without altering its transcript levels, mRNA-based stratification may fail to identify patients with high PGK1 protein activity driven by PARK2 deficiency. These findings underscore the importance of evaluating the PARK2-PGK1 axis at the protein level when considering its use as a clinical biomarker.

These findings have several important clinical and therapeutic implications. First, the inverse correlation between PARK2 and PGK1 protein levels in TNBC tissues validates the mechanistic findings and suggests that loss of PARK2 expression may be a key event allowing PGK1 accumulation in tumors. The loss of PARK2 in TNBC may be attributed to several genomic and epigenetic factors. The *PARK2* gene is located on chromosome 6q26, a region frequently targeted by loss of heterozygosity (LOH) and copy number deletions in breast cancer [51]. Additionally, epigenetic silencing via promoter hypermethylation has been identified as a frequent mechanism for *PARK2* downregulation in various tumors [52,53], potentially explaining its reduced expression in the TNBC cohorts. Given the recent discovery that PARK2 can also modulate the immune microenvironment via PD-1 degradation [46] and that PGK1 stabilization via succinylation contributes to immune escape [43], future studies should investigate whether the PARK2-PGK1 axis also influences the efficacy of immunotherapy in TNBC. Second, PARK2 can overcome PGK1-driven metastasis even when PGK1 is overexpressed suggests that enhancing PARK2 activity might be effective even in tumors with high PGK1 levels. Third, the specific PARK2-PGK1 interaction interface identified, particularly involving the RING2 domain, could serve as a template for designing small molecules that either enhance this interaction or mimic PARK2’s effect on PGK1 stability.

Several limitations of this study warrant consideration. First, while this study focused on PARK2 as a key E3 ligase for PGK1, the relative contributions of other E3 ligases in different cellular contexts remain to be determined. Second, the mechanisms underlying PARK2 downregulation in TNBC require further investigation. While deletion mutants implicated the PARK2 RING2 domain in PGK1 degradation, the use of a catalytically inactive mutant (e.g., PARK2 C431S) and mapping of specific PGK1 ubiquitination sites in the future would provide more definitive evidence. Third, the metabolic consequences of PGK1 degradation and their contribution to the anti-metastatic effects of PARK2 deserve detailed exploration. Finally, *in vivo* study relied on a tail-vein injection model. While this effectively demonstrates the ability of PARK2 to suppress lung colonization, it does not recapitulate the initial steps of the metastatic cascade. Future studies utilizing orthotopic implantation models will be necessary to fully validate the role of PARK2 in the complete metastatic process.

## 5 Conclusion

The PARK2-PGK1 axis might play a significant role in regulating partial EMT and lung colonization in TNBC. By demonstrating that PARK2 restoration promotes PGK1 degradation, this study provides a rationale for investigating therapeutic strategies that either restore PARK2 function or directly modulate PGK1 stability in aggressive breast cancers. While further research is needed to fully map the endogenous regulatory network and validate these findings in spontaneous metastasis models, this study highlights the potential of PARK2 activators or PGK1 destabilizers, potentially in combination with existing therapies, to improve outcomes for patients with TNBC.

## Data Availability

The data that support the findings of this study are available from the corresponding author upon reasonable request.
